# DNA Damage of Glioblastoma Multiform Cells Induced by Beta Radiation of Iodine-131 in The Presence or Absence of Topotecan: A Picogreen and Colonogenic Assay

**DOI:** 10.22074/cellj.2015.516

**Published:** 2015-04-08

**Authors:** Nazila Eyvazzadeh, Ali Neshasteh-Riz, Seyed Rabee Mahdavi

**Affiliations:** 1 Radiation Research Center, Faculty of Paramedicine, AJA University of Medical Sciences, Tehran, Iran; 2 Department of Radiology, Faculty of Allied Medicine, Iran University of Medical Sciences, Tehran, Iran; 3 Department of Medical Physics, Faculty of Medicine, Iran University of Medical Sciences, Tehran, Iran

**Keywords:** DNA Damage, Topotecan, Glioblastoma, Cell Death

## Abstract

**Objective:**

Glioblastoma multiforme (GBM), one of the most common and aggressive malignant brain tumors, is highly resistant to radiotherapy. Numerous approaches have been pursued to find new radiosensitizers. We used a picogreen and colonogenic assay to appraise the DNA damage and cell death in a spheroid culture of GBM cells caused by iodine-131 (I-131) beta radiation in the presence of topotecan (TPT).

**Materials and Methods:**

U87MG cells were cultured as spheroids with approximate diameters of 300 μm. Cells were treated with beta radiation of I-131 (at a dose of 2 Gy) and/ or TPT (1 μg/ml for 2 hours). The numbers of cells that survived were compared with untreated cells using a colonogenic assay. In addition, we evaluated possible DNA damages by the picogreen method. The relation between DNA damage and cell death was assessed in the experimental study of groups.

**Results:**

The findings showed that survival fraction (SF) in the I-131+TPT group (39%) was considerably less than the I-131 group (58.92%; p<0.05). The number of single strand breaks (SSB) and double strand breaks (DSB), in the DNA of U87MG cells treated with beta radiation of I-131 and TPT (I-131+TPT) significantly increased compared to cells treated with only I-131 or TPT (p<0.05). The amount of SSB repair was more than DSB repair (p<0.05). The relationship between cell death and DNA damage was close (r≥0.6) and significant (p<0.05) in the irradiated and treated groups. Also the maximum rate of DNA repair occurred 24 hours after the treatments. A significant difference was not observed on other days of the restoration.

**Conclusion:**

The findings in the present study indicated that TPT can sensitize U87MG cells to radiation and increase DNA damages. Potentially, TPT can cause an increase in damage from DSB and SSB by its inhibitory effects on topoisomerase enzyme and the cell cycle. The increased complex damages following the use of a genotoxic agent and beta I-131 radiation, causes a significant increase the cell death because of the difficult repair process. By assessing the relationship between DNA damage and cell death, the picogreen method can be useful in predicting colonogenic assay. Consequently, it is suggested that co-treatment with I-131 beta radiation and TPT can improve GBM treatment.

## Introduction

Glioblastoma multiform (GBM) is one of the most frequent, malignant primary brain tumors in adults (1,2). The approximate incidence of malignant glioma is 5 per 100,000 people worldwide, and it is more common among Americans and Africans (1,3). Due to lack of an effective treatment, there is little hope for patients with GBM. After surgery, radiotherapy is the most effective treatment strategy for these patients (4,5). GBM tumors hypoxia leads to resistance to radiotherapy, and several attempts have been made to design effective radiosensitizers (1). 

Topotecan (TPT), as a radiosensitizer agent, is a recent chemotherapy drug derived from the Chinese tree Camptotheca acuminata (6,7). TPT stops the cell cycle through inhibition of topoisomerase I (Topo-I) enzyme activity. Inhibition of the cell cycle by this enzyme will lead to DNA phosphorylation, which in turn increases both the DNATopo І complex and double strand breaks (DSB), which finally lead to apoptotic death (4,7,8). 

In order to increase the survival time for GBM patients, research has shown that the combination of low linear energy transfer radiation (LET) with TPT could result in only an increase of 1-2 years in survival (9,10). 

This issue motivates researchers to use a combination of TPT with high LET radiation. On the other hand, little information is available regarding the mechanism of changes in the cell nuclei by TPT on cancer cells of different organs. 

Recent radiotherapy and chemotherapy studies have shown that best results were achieved when TPT was injected 2-4 hours prior to radiation (11,12). Rave-Frank et al. (13) showed that combined radiation and TPT treatment lead to reduction of colonogenic cell survival in glioblastoma cells. McCluskey et al. (14) reported that simultaneous administration of PJ34 and (131) I-meta-iodobenzylguanidine/TPT [(131) I-MIBG/TPT] induced supraadditive toxicity in noradrenaline transporter (NAT) transfected glioma cells. 

Iodine-131 (I-131) is one of the radioisotopes which emits beta and gamma rays. The principal radiant that causes damage is the beta ray. Beta rays have more ionizing power in comparison with the X photon. Their prominent advantage in cellular damage is based on crossfire phenomenon; in which rays with several cell depths transfer energy to neighboring and distant cells (cross-dose) in addition to creating self-dose in surrounding cells. This phenomenon increases DNA breaks. If the cancer cells receive less self-dose, it will be compensated by a cross-dose (15). This is particularity important in tumor treatment and has been widely used in the treatment of thyroid and central nervous system (CNS) tumors (16). Previous experimental study has also shown that the combination of chemotherapeutic agents with high-LET particle beams can enhance the cellular effect that is comparable to photon irradiation (17). 

Direct or indirect interaction of ionizing radiation induces a variety of DNA damage such as single strand breaks (SSB), DSB, base damage (BD) of various types and DNA-protein crosslinks (18). We have used the picogreen method as a suitable tool to determine the single strand DNA (ssDNA)/ double strand DNA (dsDNA) ratio and a sensitive probe for determing the level of damage to dsDNA. The fast micromethod was introduced by Batel et al. (19) and subsequently modified for analyzing high number of samples in a short time. 

The colonogenic assay is also appropriate to ensure that radiation-induced DNA damage leads to cell death. Although numerous papers have reported the relationship between colonogenic radiosensitivity, radiation induced apoptosis and DNA damage, other methods such as the comet assay and γH2AX are also used (20,21). 

The primary objective of this study was to investigate the impact of TPT co-treatment with I-131 beta radiation therapy on DNA damage, repair of damage and cell death in GBM cells using the picogreen and colonogenic assays. This study also compared these two techniques. 

## Materials and Methods

### Cell line

The human GBM cell line U87MG was purchased from Pasteur Institute of Iran. This cell line was cultured in minimum essential medium (MEM; Gibco/Invitrogen, USA) that contained 10% fetal bovine serum (FBS; GmbH/ PAA, Austria), 100 U/ml of penicillin streptomycin (GmbH/ PAA, Austria) and 20 U/ml of fungizone (Gibco/ Invitrogen, USA). 

### Monolayer culture

In the experimental study, cells were cultured as a monolayer at a density of 25×10^4^cells/cm^2^in T-25 tissue culture flasks (NEST).Cultures were maintained at 37˚C in a humidified atmosphere and 5% CO_2_. Cells were harvested by trypsinizing cultures with 0.25% trypsin (Sigma/Aldrich, Germany) and 0.03% ethylenediaminetetraacetic acid (EDTA; Sigma/Aldrich, Germany) in phosphatebuffered saline (PBS; MP Biomedicals, Germany). 

### Spheroid culture

Spheroids were cultured using the liquid overlay technique. A total of 5×10^5^cells were seeded into NEST coated with a thin layer of 1% agar (Sigma/ Aldrich, Germany) with 10 ml of MEM supplemented with 10% FBS. The plates were incubated at 37˚C in a humidified atmosphere and 5% CO_2_(Memmert, Germany). Half of the culture medium was replaced with fresh culture medium every three days. 

### Growth curve

After three passages of monolayer culture, the cells were cultured at a density of 10000 per well in multiwell plates (24 wells/plate; Greiner). The multiwall plates were incubated at 37˚C in a humidified atmos phere and 5% CO_2_. For a nine days period, at 24 hour intervals, the cells from the triplicate wells were removed with 1mM EDTA/0.25% trypsin (w/v) treatment and counted in a hemocytometer. An average of nine counts was used to define each point [mean ± standard error mean (SEM)]. Half of the culture medium was replaced with fresh medium twice per week. We plotted a growth curve where in the linear area or logarithmic phase of the curve, we calculted the cell numbers as follows: N=N0×e^bt^, in which "N0" is the initial number of the cells, "N" is the number of the cells after time, "t" and "b" shows the gradient of the logarithmic phase of the curve. Then, the population doubling time of the cells was determined according to the gradient of the logarithmic phase of the curve. 

To draw the spheroid growth curve, one spheroid cell was seeded per well in multi-well plates coated with a thin layer of 1% agar with 1 ml of MEM. The multi-well plates were incubated at 37˚C in a humidil fied atmosphere and 5% CO_2_. For 28 days, at 72 hour intervals, we measured the vertical diameters of the cells by a microscope. The measurements were performed in triplicate. Next, the cell volume were calculated according to the formula V=a.b^2^.π/6, where "a" is the small diameter of the cells, "b" is the large diameter of the cells and "V" shows the volume of the spheroid cells. An average of nine counts was used to define each point (mean ± SEM). Half of the culture medium was replaced with fresh medium twice per week. Next, we plotted the growth curve, where in the linear area or logarithmic phase of the curve, we calculated the volume of cells as follows: V=V_0_× e^kt,^in which "V_0_" is the initial volume of the cells, "V" is the volume of cells after time, "t" and "k" shows the gradient of the logarithmic phase of the curve. Then, the volume doubling time (VDT) of the cells was determined according to the gradient of the logarithmic phase of the curve. 

### Drug treatment and beta cell irradiation by I-131

The GBM cells were grown on a layer as three dimensional spheroid cells (diameters: approximately 300 μm) in a liquid method. We divided the cells into four groups: i. control, ii. TPT: cells treated with 1 μg/ ml of TPT for 2 hours, iii. I-131: cells incubated with a solution of 10 mci (millicurie) I-131 in 0.2 M NaOH for 108 minutes and iv. I-131+TPT: cells incubated with a solution of 10 mci I-131 in 0.2 M NaOH for 108 minutes after which they were treated with TPT for 2 hours. The flask was exposed for 108 minutes to determine the correlation between DNA damage and the absorbed dose of 2 Gy (22). 

Subsequently the flasks that contained medium were centrifuged, then cells washed and centrifuged twice with PBS to remove the I-131. At the end of the exposure and treatment periods, we evaluated DNA damage by the picogreen method. Groups three and four were assessed for DNA damage daily for seven days after the treatments. The colonogenic ability of cells was evaluated by colony assay in groups one, three and four. 

### Picogreen assay

The picogreen assay is an easy, rapid, and sensitive micromethod that determines the extent of DNA damage (DSB, SSB) in individual cells, induced by a variety of genotoxic agents (22,23). We used the picogreen assay to evaluate radiation-induced SSB and DSB damages in the DNA of GBM cells according to a protocol as previously mentioned by Schroder et al. (24). The solutions used are as follows. A fluorescent dye stock solution was the picogreen dsDNA quantitation reagent (solution A; Life Technology/ Invitrogen, USA). Calcium (Ca) and magnesium (Mg) free PBS (Ca/Mg free PBS) consisted of 137 mM NaCl, 2.7 mM KCl, 4.3 mM Na_2_HPO_4_and 1.5 mM KH_2_PO_4_(solution B; Gibco/Invitrogen, USA). The lysing solution contained 9.0 M urea, 0.1% sodium dodecyl sulfate (SDS) and 0.2 M EDTA at a pH of 10 with NaOH (solution C; Sigma/Aldrich, Germany). Lysing solution supplemented with picogreen consisted of 10 μL of the original stock dye/ml of solution C (solution D; Life Technology/Invitrogen, USA). Solution E (Sigma/Aldrich, Germany) consisted of 20 mM EDTA and solution F (Gibco/Invitrogen, USA) consisted of an NaOH stock solution (1.0 M NaOH and 20 mM EDTA). The working NaOH solution was prepared fresh prior to use. A total of 2 mL of solution F was added to 18 mL of solution E with a pH of 12.40 ± 0.02 to create solution G. 

In order to determine the DSB induced in GBM cells, 3 falcon pipes that consisted of 50,000 cells/ mL with 300 µl of solution C and 300 µl of solution D. in order to lyse the cells, these groups were placed in the dark for 40 minutes. The amount of DSB was determined after 40 minutes by using a 485 nm excitation wavelength and 528 nm emission wavelength. 

Next, 50 μl of solution G was added to 600 μl of the lysed cells in each group (control, irradiated and treated+irradiated). The amount of SSB were determined after three hours of incubation by measuring the fluorescence intensity of each group. 

### Calibration curve

The various concentrations of DNase with 300 μl of solution D and a given volume of PBS (final reaction volume: 800 µL) were added to various concentrations of non-irradiated GBM cells. Next, the amount of fluorescence intensity for digestion DNA in each group was measured after three hours of incubation. 

### Colonogenic assay

The colonogenic assay determines cell death following radiation (25). We evaluated the colonogenic ability and surviving fraction of GBM cells by the colony assay according to the manufacturer’s protocol by Franken et al. (26). Treated cells from each of the groups were seeded at the appropriate concentrations into 25 cm^2^flasks. Colonies are fixed with formaldehyde (Gibco/Invitrogen, USA) 0.2% v/v for five minutes, stained with crystal violet (0.5% w/v) for forty minutes, and counted using an optical microscope (Techno/Meiji, Japan). Colonies were defined as cell aggregates the approximate number of which was more than 50. We calculated the number of colonies, plating efficiency (PE) and survival fraction (SF). The PE is the ratio of the number of colonies to the number of cell seeds. Colonogenic efficiency is the SF, which is defined as the PE in treated cells divided by the PE in untreated cells. The SF was calculated after determining PE. 

### Statistical analysis

Statistical analysis was performed using the independent-samples t test and one-way analysis of variance (ANOVA) followed by the scheffe test as the post-hoc analysis using statistical package for the social sciences (SPSS) version 16. Pearson’s correlation coefficient was used to determine the relationship between cell death and DNA damage. P<0.05 was considered to be significant. All values are expressed as mean ± S.E.M for all tests. 

## Results

### Cell characteristics

#### Monolayer culture

**Fig.1:**
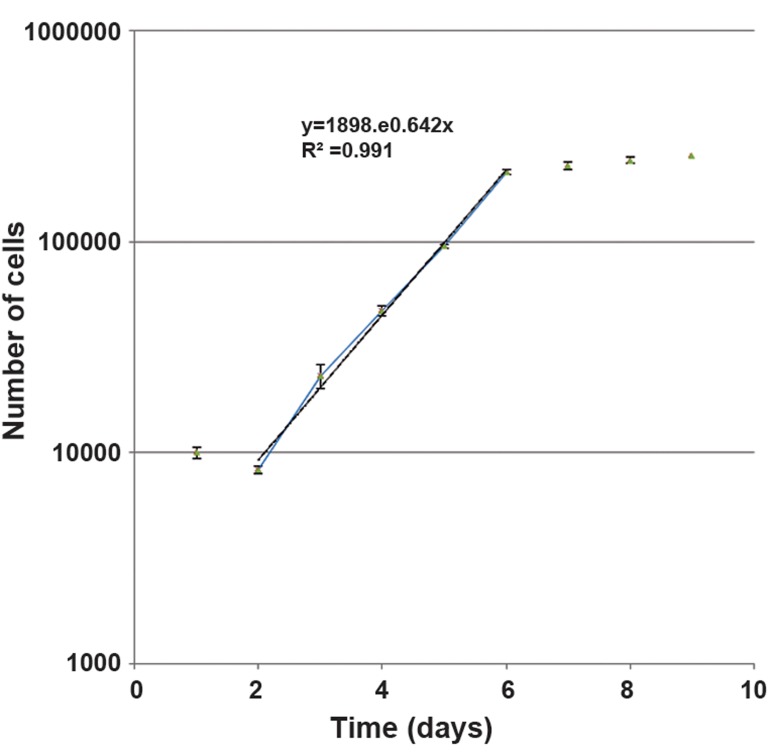
Growth curve of U87MG cell line in the monolayer cultures. An average of nine counts was used to define each point. Mean ± SEM of three experiments. Y; Number of cell, X; Time (day), R; Regression coefficient and SEM; Standard error of the mean.

The U87MG GBM cell line grows as a monolayer in the tissue culture flasks. The growth curve of these cells in the monolayer culture is shown in figure 1. The population doubling time calculated from this curve was approximately 25.9 ± 0.39 hours (Fig.1). 

#### Spheroid cult Spheroid culture

**Fig.2:**
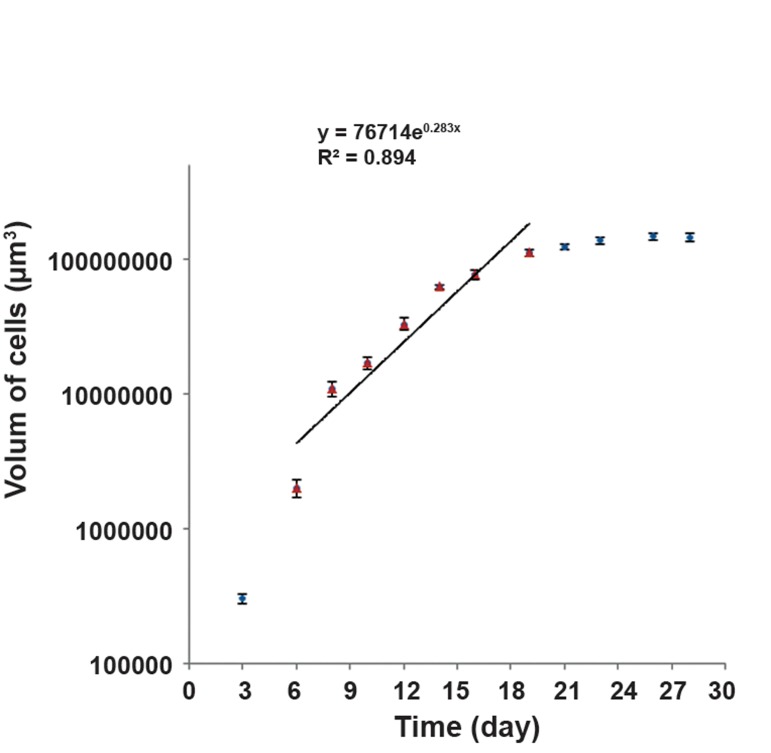
Growth curve of U87MG cell line in spheroid Cultures. An average of nine counts was used to define each point. Mean ± SEM of three experiments is shown on the curve. Y; Volume (μm3), X; Time (day), R; Regression coefficient and SEM; Standard error of the mean.

The U87MG cells formed spheroids in the liquid overlay cultures. The volume doubling time of these spheroids is approximately 58.77 hours (Fig.2). 

### DSB and SSB

The picogreen assay was used to determine the extent of SSB and DSB damage. The spectrum was derived from samples of the irradiated, treated and control groups of U87MG cells of 300 µm spheroids. We used the spectroflourometer (Shimadzu, Japan) at an excitation wave length of 485 nm and eoups. This reduction showed DSB and SSB damages in irradiated and treated GBM cells. 

**Table 1:** The measured average fluorescence intensities of control glioblastoma (GBM) cells and those irradiated by 2 G y of beta iodine-131, (I-131) to determine the amount of single-strand breaks (SSB) and double-strand breaks (DSB) in presence topotecan (TPT)


Group	Fluorescence (DSB) (nm)	Fluorescence (SSB) (nm)

**Control**	595.72 ± 3.32	496.64 ± 3.50
**TPT**	567.00 ± 3.20	482.00 ± 2.32
**I-131**	490.00 ± 1.74	428.50 ± 2.50
**I-131+TPT**	455.00 ± 2.32	411.00 ± 2.81


Data are presented as mean ± standard error of the mean (SEM).

**Fig.3:**
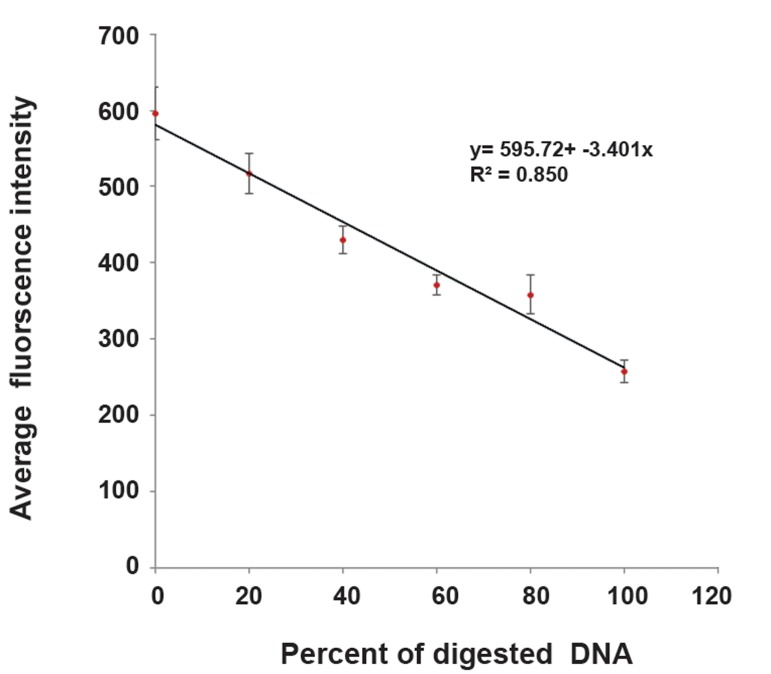
Calibration curve obtained by the amount of fluorescence intensities of the control group and DNasetreated grousps. 3.4 changes in amount of fluorescence intensity indicated a 1% break in DNA. Data are presented as mean ± SEM. Y; Fluorescence intensity, X; Percent of digestion, R; Regression coefficient and SEM; Standard error of the mean.

A calibration curve was drawn to measure SSB and DSB in the cell group. To plot the curve, the average amounts of fluorescence intensity were derived from the control and treated groups. The average fluorescence intensity in the control group in compared to the treated groups was significantly differed (p<0.05). A gradient of the linear phase of the curve showed a 1% break in DNA. The difference of intensity per break in the DNA strand was 341.5, which meant that for each 3.415 change in amount of fluorescence intensity, a 1% break would occur in the DNA strand. The calibration curve is shown in figure 3.

**Fig.4:**
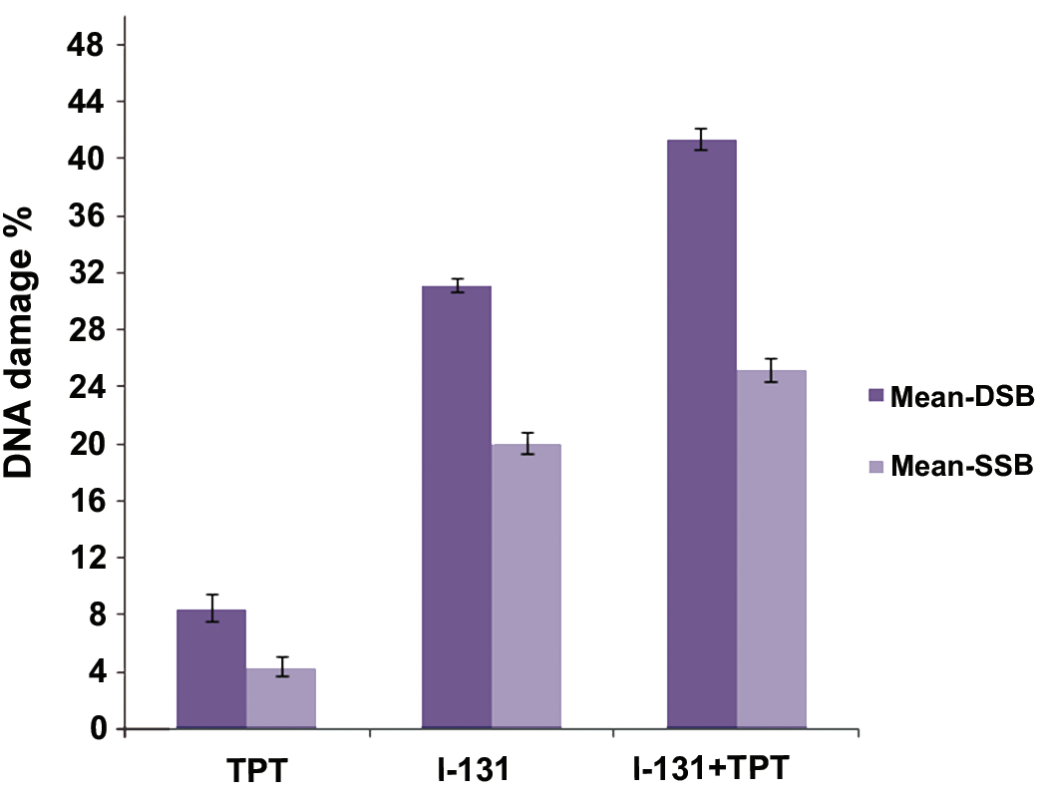
Distribution of the average percentage of single-strand breaks (SSB) and double-strand breaks (DSB) of glioblastoma (GBM) cells in groups irradiated (2Gy of beta I-131) and treated with topotecan (TPT). Data are presented as mean ± standard error of the mean (SEM).

The difference between average intensities in the control and treated groups was divided by 3.4 to determine the percent of SSB and DSB damage (Fig.4). 

Figure 4 shows that I-131+TPT had significantly increased DSB and SSB damage in compared to the I-131 and TPT groups (p<0.05). 

### Reduction in DSB and SSB

**Fig.5:**
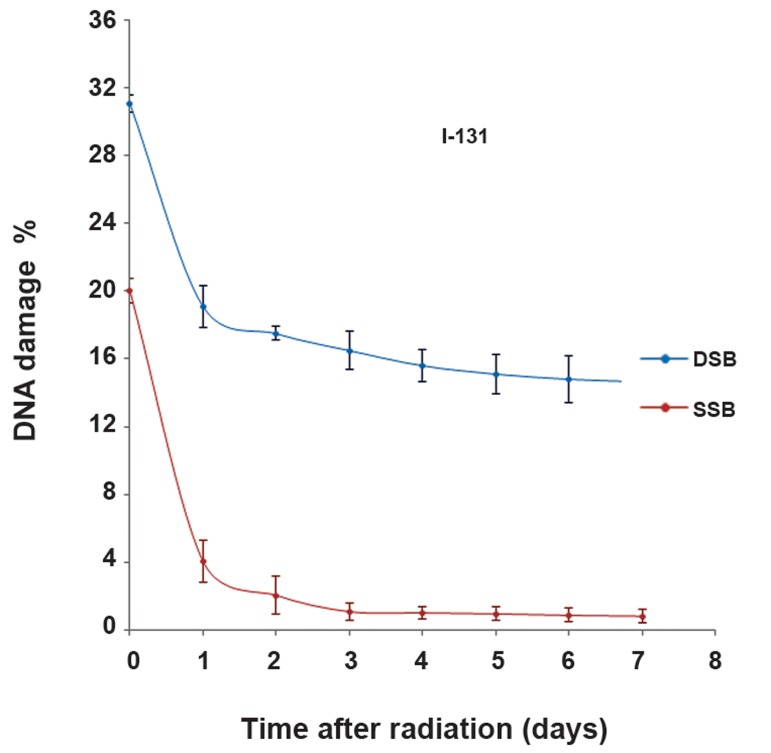
Distribution of the average percentage of single-strand (SSB) and double-strand (DSB) of glioblastoma (GBM) cells in groups irradiated with 2Gy of beta iodine-131 (I-131) for 7 days after radiation. Data are presented as mean ± standard error of the mean (SEM).

**Fig.6:**
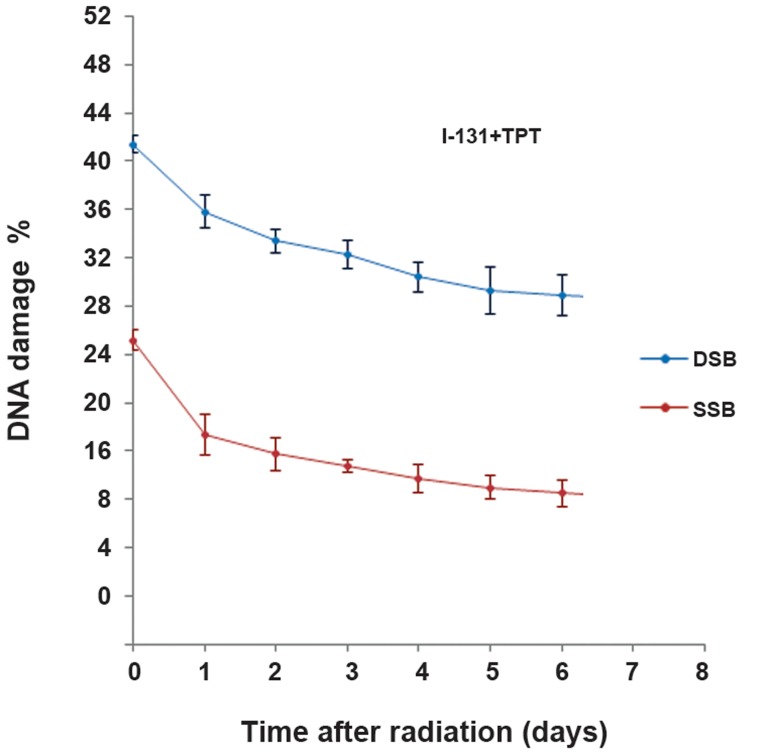
Distribution of the average percentage of single-strand breaks (SSB) and double-strand breaks (DSB) of glioblastoma (GBM) cells in groups irradiated with2Gy of beta iodine (I-131) and treated with topotecan (TPT) after 7 days of radiation and treatment. Data are presented as mean ± standard error of the mean (SEM).

In order to determine the amount of repair, we evaluated the DNA damage for a seven day period at 24 hour intervals after radiation and treatment by using the picogreen assay. Figures 5 and 6 show the reduction DNA damage in the I-131 and I-131+TPT groups for 7 days after treatment. A decrease in the amount of DNA damage indicated the amount of repair in the I-131 irradiated groups in the presence or absence of TPT. The maximum rate of DNA repair occurred 24 hours after irradiation and treatment. A significant difference was not observed on other days of the restoration. The amount of SSB repair was more than the amount of DSB repair. The amount of restoration of DNA damage in the I-131 group was greater than the I131+TPT group. 

### PE and SF

**Fig.7:**
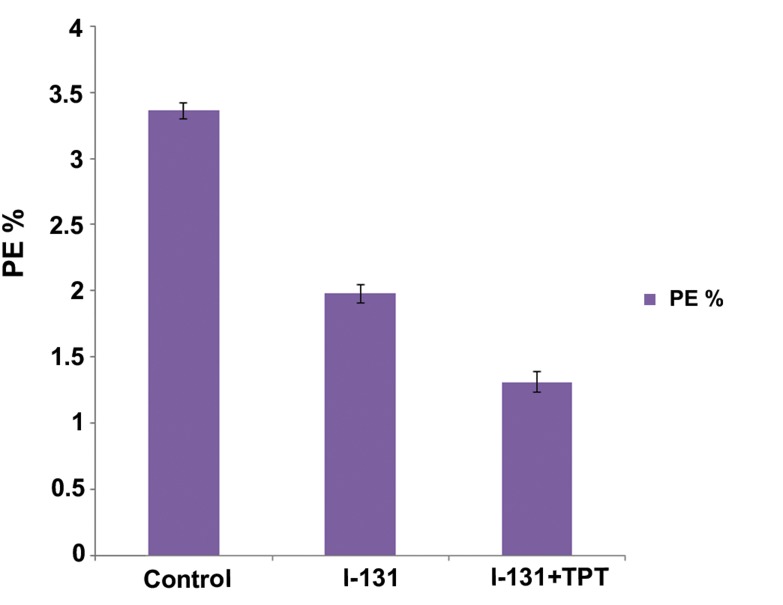
Plating efficiency (PE) for glioblastoma (GBM) cell line in control, iodine131 (I-131)+topotecan (TPT) and I-131 groups. Data are presented as mean ± standard error of the mean (SEM).

**Fig.8:**
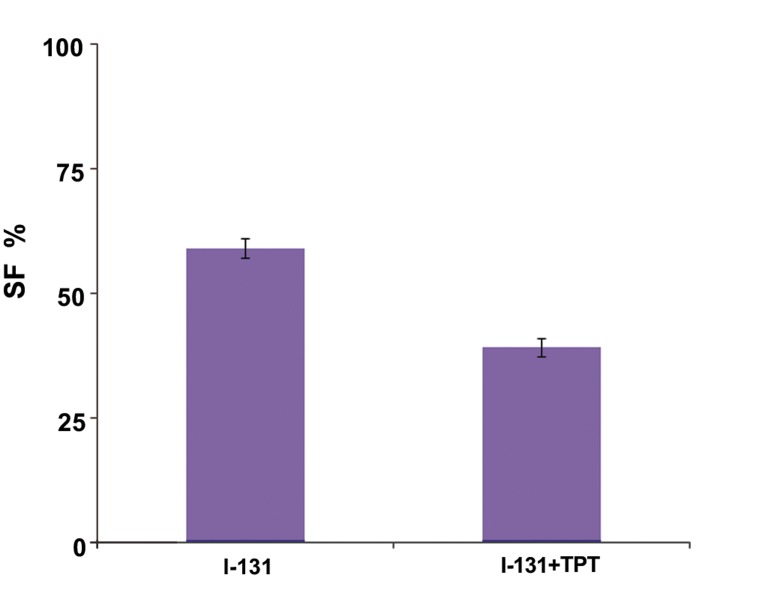
Fraction of cells that survived for the glioblastoma (GBM) cell line in the iodine-131 (I-131)+topotecan (TPT) and I-131 groups. Data are presented as mean ± SEM. SEM; Standard error of the mean and SF; Survival factor.

The average numbers of PE efficiency for the GBM cell line in the control group are shown in table 2. Maximum PE was observed when 5000 cells were seeded into flasks (3.36%). The fraction of cells that survived and PE in irradiated and treated groups are shown in figures 7 and 8. In figure 7, the I-131+TPT group had a decreased percentage of PE compared with the I-131 group (p<0.05). Furthermore, cell death significantly increased in the presence of I-131+TPT compared with the I-131 group (p<0.05). As seen in figure 8, SF in the I131+TPT group (39%) was less than I-131 group (58.92%). 

### Correlation between DNA damage and cell death

In table 3, the Pearson correlation coefficient analysis revealed that the relationship between cell death and DNA damage was close (r≥0.6) and significant (p<0.05) in the irradiated and treated groups. 

**Table 2:** Average number of colonies and plating efficiency (PE) for glioblastoma GBM) cell line in the control group


Cell number	Colonies	PE (%)

**3000**	75 ± 5.42	2.5 ± 0.18
**4000**	112 ± 6.80	2.8 ± 0.17
**5000**	168 ± 3.00	3.36 ± 0.06
**6000**	197 ± 5.40	3.28 ± 0.09
**7000**	223 ± 4.94	3.18 ± 0.07


Data are presented as mean ± standard error of the mean (SEM).

**Table 3:** Comparison of cell death and DNA damage in glioblastoma (GBM) cells in the iodine-131 (I-131) and I-131+topotecan (TPT) groups according to the colonogenic and picogreen assays and their correlation (p<0.05)


Group	Mean DNA damage (%)	Mean cell death (%)	Pearson coefficient (r)	P value

**I-131**	51.14	41.08	0.91	0.040
**I-131+TPT**	66.58	61	0.97	0.037


## Discussion

TPT has a commercial name of Hycamtin. It is a radiosensitizer and one of the more recent chemotherapy drugs used for studies both *in vitro* and *in vivo*. TPT is derived from camptothecin (8, 11, 27). This compound is used as treatment for a vast array of cancers such as ovarian, lung, leukemia, non-Hodgkin’s lymphoma, myelodysplastic syndrome, melanoma and colorectal cancers. Recently, research is being performed on GBM in children and adults (8). TPT inhibits the enzyme Topo-I and binds to DNA to form an isomerase DNA complex and aggregation of the stabilized complex which leads to SSB and DSB damage and finally cell death (28).

Scientific erosion and experimental study have shown an increased complexity and severity of complex DNA damage with increasing LET (29). High-LET radiation can produce up to 25 damages compared with 10 per cluster in low-LET radiation (30). Complex DNA damages are difficult to repair, and may lead to cell death (31).

The purpose of the current study was to apply picogreen assay as an evaluation of DNA damage and colonogenic assay to assess cell death caused by I-131 in the presence of TPT. We used 300 μm diameter spheroid cultures of the human GBM cell line U87MG. Ultimately, these two techniques would compare the relation between DNA damage and cell death.

Our results showed significantly increase DSB and SSB damages in the presence of TPT after beta radiation with I-131 compared with only I-131. Cell death also significantly increased after radiation with I-131 and incubation with TPT for two hours as compared with the I-131 group. Using TPT which inhibits the Topo-I enzyme and forms a complex on the DNA (32), we observed a higher level of DNA damage induced by radiation with I-131 after incubation with TPT. The relation between DNA damage and cell death was closer in the I-131+TPT group (1.09) compared to the I-131 group (1.24). Therefore, we concluded that the picogreen method was useful for predicting colonogenic assay following exposure to genotoxic agents and beta irradiation with I-131. This could be due to increasingly complex DNA damage, particularly DSB damage in the presence of genotoxic agents and high LET radiation of beta I-131.

Previous study has reported that up to 90% of complex DSB are created by high LET radiation; whereas 30% of those are created by low LET radiation (31). On the other hand, repair of complex DNA damage rarely occurs, rather there is cell death and the formation of chromosome aberrations (33, 34). Barazzuol et al. (35) have demonstrated that high LET radiation combined with temozolomide (TMZ) had an enormous potential for treating a radioresistant tumor such as GBM.

Banath et al. (20) reported that residual γH2AX predicted colonogenic fraction subsequent to exposure to cisplatin, TMZ and camptothecin (CPT) drugs in SiHa cervical cancer cells.

The current study results revealed that the percentage of DSB (12%) and SSB (16%) repairs were greater in the I-131 group compared to the I-131+TPT group (DSB: 7.50%) (SSB: 10.49%) at 24 hours after treatment. Since DNA repair mechanisms were disturbed in the I-131+TPT group, the resultant damages were very severe. Our results indicated that the repair of DSB was (12%) at 24 hours after irradiation with 2 Gy in GBM cells (36).

Chu et al. (37) observed that repair of DSB was (10%) at 6 hours after irradiation with 2 Gy the presence of BO-1051 in cell line U87MG. Ma et al. (38) reported that the repair of DSB was less than the repair of SSB in a Raji cell line after γirradiation with 100 Gy using epstein-barr virus (EBV).

## Conclusion

Treatment of cells with TPT after I-131 beta radiation has significantly increased complex DNA damage and may improve the therapeutic index for radiation. Our purpose for further studies is to use TPT liposomes modified with tamoxifen (TAM) and wheat germ agglutinin (WGA) to improve drug transport across the blood-brain barrier, develop drug stabilization, and subsequently evaluate the combined effects of these agents on cells.

## References

[1] Van Meir EG, Hadjipanayis CG, Norden AD, Shu HK, Wen PY, Olson JJ (2010). Excitting new advances in neuro-oncology: the avenue to a cure for maliggnant glioma. CA Cancer J Clin.

[2] Squtrito M, Holland EC (2011). DNA damage response and growth factor signaling pathways in gliomagenesis and therapeutic resistanc. Cancer Res.

[3] Stupp R, Tonn JC, Brada M, Pentheroudakis G (2010). ESMO Guidelines Working Group.High-grade malignant glioma: ESMO Clinical Practice Guidelines for diagnosis,treatment and follow-up. Ann Oncol.

[4] McCluskey AG, Boyd M, Pimlott SL, Babich JW, Gaze MN, Mairs RJ (2008). Experimental treatment of neuroblastoma using [131I]meta-iodobenzylguanidine and topotecan in combination the british. Br J Radiol.

[5] Brandes AA, Compostella A, Blatt V, Tosoni A (2006). Glioblastoma in the eldery: current and future trends. Crit Rev Oncol Hematol.

[6] Bernier-Chastagner V, Grill J, Doz F, Bracard S, Gentet JC, Marie-Cardine A (2005). Topotecan as a radiosensitizer in the treatment of children with malignant diffuse brainstem gliomas: results of a French Society of Paediatric Oncology Phase II Study. Cancer.

[7] Tomicic MT, Chritmann M, Kaina B (2005). Topotecan-triggered degradation of topoisomerase I is p53-dependent and impacts cell survival. Cancer Res.

[8] Stathopoulos GP, Ardavanis A, Papakotoulas P, Pectasides D, Papadopoulos G, Antoniou D (2010). Myelotoxicity of oral topotecan in relation to treatment duration and dosage:a phase I study. Anticancer Drugs.

[9] Gross MW, Altscher R, Brandtner M, Hausser-Mischlich H, Kiricuta IC, Siegmann AD (2001). Acute toxicity and changes in quality of life during a combined radio-chemotherapy of glioblastomas with topotecan (Hycamtin). Strahlenther Onkol.

[10] Gross MW, Altscher R, Brandtner M, Haeusser-Mischlich H, Chiricuta IC, Siegmann AD (2005). Open-lable simultaneous radio-chemotherapy of glioblastoma multiforme with topotecan in adults. Clin Neurol Neurosurg.

[11] Koster DA, Palle K, Bot ES, Bjornsti MA, Dekker NH (2007). Antitumor drugs impede DNA uncoiling by topoisomerase I. Nature.

[12] Fisher B, Won M, Macdonald D, Johson DW, Roa W (2002). Phase П study of topotecan plus cranial radiation for glioblastoma multiforme: results of radiation therapy oncology grup 9513. Int J Radiat Oncol Biol Phys.

[13] Rave-Frank M, Glomme S, Hertig J, Weiss E, Pradier O, Hess CF (2002). Combined effect of topotecan and irradiation on the survival and the induction of chromosome aberrations invitro. Strahlenther Onkol.

[14] McCluskey AG, Mairs RJ, Tesson M, Pimlott SL, Babich JW, Gaze MN (2012). Inhibation of poly(ADP-Ribose) polymerase enhances the toxicity of 131Imetaiodobenzylguanidine/topotecan combination therapy to cells and xenografts that express the noradrenaline transporter. J Nucl Med.

[15] Enger SA, Hartman T, Carlsson J, Lundqvist H (2008). Cross-fire doses from beta-emitting radionuclides in targeted radiotherapy.A theoretical study based on experimentally measured tumor characteristics. Phys Med Biol.

[16] Goldsby RE, Fitzgerald PA (2008). Meta[I131]iodobenzylguanidine therapy for patients with metastatic and unresectable pheochomocytoma and paraganglioma. Nucl Med Biol.

[17] Combs SE, Zipp L, Rieken S, Habermehl D, Brons S, Winter M (2012). Invitro evaluation of photon and carbon ion radiotherapy in combination with chemotherapy in glioblastoma cells. Radiat Oncol.

[18] Sutherland BM, Bennett PV, Sidorkina O, Laval J (2000). Clustered DNA damages induced in isolated DNA and in human cells by low doses of ionizing radiation. Proc Natl acad Sci USA.

[19] Batel R, Jaksic Z, Bihari N, Hamer B, Fafandel M, Chauvin C (1999). A microplate assay for DNA damage determination (fast micromethod). Anal Biochem.

[20] Banath JP, Klokov D, MacPhail SH, Banuelos CA, Olive PL (2010). Residual gammaH2AX foci as an indication of lethal DNA lesions. BMC Cancer.

[21] Dunne AL, Price ME, Mothersill C, McKeown SR, Roson T, Hirst DG (2003). Relationship between clonogenic radiosensitivity radiation-induced apoptosis and DNA damage/repair in human colon cancer cells. Br J Cancer.

[22] Heng BC, Das GK, Zhao X, Ma LL, Tan TT, Ng KW (2010). Comparative cytotoxicity evaluation of lanthanide nanomaterials on mouse and human cell lines with metabolic and DNA-quantification assays. Biointerphases.

[23] Ashley N, Harris D, Poulton J (2005). Detection of mitochondrial DNA depletion in living human cells using picoGreen staining. Exp Cell Res.

[24] Schroder HC, Batel R, Schwertner H, Boreiko O, Muller WE (2006). Fast micromethod DNA single-strand-break assay. Methods Mol Biol.

[25] Wouters A, Pauwels B, Lambrechts HA, Pattyn GG, Ides J, Baay M (2010). Counting clonogenic assays from normoxic and anoxic irradiation experiments manually or by using densitometric software. Phys Med Biol.

[26] Franken N, Rodermond HM, Stap J, Haveman J, van Bree C (2006). Clonogenic assay of cells in vitro. Nat Protoc.

[27] Vassal G, Pondarre C, Capelli C, Terrier-Lacombe MJ, Boland I, Morizet J (1997). DNA-topoisomerase I, a new target for the treatment of neuroblastoma. Eur J Cancer.

[28] Pinel S, Chastagner P, Merlin JL, Marchal C, Taghian A, Barberi-Heyob M (2006). Topotecan can compensate for protracted radiation treatment time effects in high grade glioma xenografts. J Neurooncol.

[29] Hada M, Georgakilas AG (2008). Formation of clustered DNA damage after high-LET irradiation: a review. J Radiat Res.

[30] Semenenko VA, Stewart RD (2004). A fast monte carlo algorithm to simulate the spectrum of DNA damages formed by ionizing radiation. Radiat Res.

[31] Eccles LJ, Lomax ME, ONeill P (2010). Hierarchy of lesion processing governs the repair, double-strand break formation and mutability of three-lesion clustered DNA damage. Nucleic Acids Res.

[32] Wang LH, Pfister TD, Parchment RE, Kummar S, Rubinstein L, Evrard YA (2010). Monitoring drug-induced gammaH2AX as a pharmacodynamic biomarker in individual circulating tumor cells. Clin Cancer Res.

[33] van den Aardweg GJ, Naus NC, Verhoeven AC, De Klein A, Luyten GP (2002). Cellular radiosensitivity of primary and metastatic human uveal melanoma cell Lines. Invest Ophthalmol Vis Sci.

[34] Iliakis G, Wang H, Perrault AR, Boecker W, Rosidi B, Windhofer F (2004). Mechanisms of DNA double strand break repair and chromosome aberration formation. Cytogenet Genome Res.

[35] Barazzuol L, Jena R, Burnet N, Jeynes JC, Merchant MJ, Kirkby KJ (2012). In vitro evaluation of combined temozolomide and radiotherapy using x rays and highlinear energy transfer radiation for glioblastoma. Radiat Res.

[36] Short SC, martindale C, Bourne S, Brand G, Woodcock M, Johnston P (2007). DNA repair after irradiation in glioma cells and normal human astrocytes. Neuro Oncol.

[37] Chu PM, Chiou SH, Su TL, Lee YJ, Chen LH, Chen YW (2011). Enhancement of radiosensitivity in human glioblastoma cells by the DNA N-mustard alkylating agent BO-1051 through augmented and sustained DNA damage response. Radiat Oncol.

[38] Ma W, Halweg CJ, Menendez D, Resnick MA (2012). Differential effects of poly (ADP-ribose) polymerase inhibition on DNA break repair in human cells are revealedwith Epstein-Barr virus. Proc Nalt Acad Sci USA.

